# Shared-decision-making, trust in the healthcare system and health literacy are associated with self-reported pain levels: a population-based cross-sectional study in individuals living with a chronic health condition in Wales

**DOI:** 10.1186/s12913-025-13724-3

**Published:** 2025-12-01

**Authors:** Katherine E. Woolley, Nichola Thuvesholmen, Sarah Puntoni, Sally Cox, Alexander Shaw, Owen Hughes, Kathleen Withers

**Affiliations:** 1 CEDAR, Cardiff and Vale Health Board, Cardiff, UK; 2 Digital Health and Care Wales, Cardiff, UK; 3 Value Transformation, NHS Wales Performance and Improvement, Wales, UK; 4 Powys Living Well Service, Powys Teaching Health Board, Bronllys, UK; 5 Cardiff University, Cardiff, UK

**Keywords:** Patient-reported outcomes measures, Patient-reported experience measures, Shared decision-making, Pain management

## Abstract

**Background:**

Pain is an invisible condition which has significant morbidity, mortality and healthcare system burden, and is a leading cause of disability worldwide. Pain is associated with negative health, health-system and social-economic consequences. Wales has a high prevalence of chronic pain and long waiting lists for chronic pain treatments. With the recommendation by NICE for person-centred care and the Welsh Government’s A Healthier Wales and Value-Based Healthcare agendas, this study aims to assess the association between self-reported pain levels and (i) experience of shared-decision-making, (ii) perception of the healthcare system, and (iii) health literacy in adults aged 46 years old and over within Wales.

**Methods:**

The nationally representative Population Health Survey collected patient-reported outcomes, experiences and basic demographics between July-October 2023 across Wales. The survey was deployed to adults aged 46 years old and over, online, using paper and via telephone. An adjusted linear regression, controlling for individuals and situational characteristics, was used to assess the association between self-reported pain levels in the last 7-days and (i) experience of shared-decision-making, (ii) level of trust in the healthcare system, and (iii) health literacy.

**Results:**

A lack of shared-decision-making, lower health literacy level and lower trust in the healthcare system were all associated with an increase in self-reported pain levels. Being female, having two or more chronic health conditions, being unable to work due to sickness or ill-health, a lower education level, not drinking alcohol, lower exercise levels and poorer mental health were all individual factors associated with an increase in self-reported pain. Living in an area with a lower deprivation level was the only situational factor associated with an increase in self-reported pain.

**Conclusion:**

Increasing shared-decision-making, health literacy levels and trust in the healthcare system are modifiable factors that could have positive health outcomes in the management of chronic pain within adults aged 46 years old and over in Wales. These results have implications for the equitable future delivery of pain and chronic health conditions service within Wales, ensuring that any service meets the Welsh Government’s Value-Based Healthcare approach, by delivering person-centred care.

**Supplementary Information:**

The online version contains supplementary material available at 10.1186/s12913-025-13724-3.

## Introduction

Pain is one of the leading disabilities worldwide [1], causing significant morbidity, mortality and healthcare system burden [2]. In Wales, it is estimated that mild to debilitating pain is experienced by 11–20% of the adult population [3, 4], with an increasing prevalence with age [5]. Pain is an invisible condition with a broad aetiology, that can either be acute (pain with sudden onset that is time limited) [6] or chronic (pain that persists or recurs for more than 3 months). Pain may be classified as primary (a standalone condition) or secondary (as a result of a comorbidity) [7].

Chronic pain can have negative health (e.g., mental health, quality of life, cardiovascular health, sexual function, cognitive processes, sleep) [8], health-system (e.g., prescription costs, healthcare usage) [9–11] and socio-economic consequences (e.g., work-force participation, work productivity, opioid dependence) [10–12]. Risk factors for chronic pain can be biological (e.g., age, sex, comorbidities, ethnicity, genetic), behavioural (e.g., smoking, drinking, inactive lifestyle, attitudes and beliefs about pain) and societal (e.g., social exclusion, socio-economic status, occupation) [13–16]. These risk factors show that the perception, expression, and impact of chronic pain [17] are linked to the social determinants of health. Therefore, if chronic pain is not adequately managed or treated, there is a risk of widening health inequalities and inequities.

NICE guidelines recommend a person-centred assessment when dealing with chronic pain [18], specifically enabling patients to actively participate in their care. Shared-decision-making, trust in the healthcare system and health literacy are all factors that enable active participation [19, 20], which can reduce the experience of pain by enabling better self-management, adoption of coping strategies and adherence to treatments [21]. Shared decision-making has been shown to reduce the experience of chronic pain [22] and plays a vital role in facilitating the reduction of opioid dependence [23]. Furthermore, integrated multidisciplinary approaches can be effective and cost- efficient in managing chronic pain compared to non-multidisciplinary care, but due to their high cost and requirement to provide longitudinal care these approaches are difficult to implement [24].

In Wales there is a need to improve chronic pain services, with a Government integrated impact assessment in 2019 stating there are very long chronic pain waiting lists [3].Wales is developing local and national initiatives to increase shared-decision making across chronic pain (e.g., specialist chronic pain services using the biopsychosocial model of assessment, upskilling staff) and long-term condition (e.g., living well services, online information, use of decision aids) services, as well as actively attempting to reduce the reliance on opioid medications, to prevent long-term addiction and harm [25]. Furthermore, a recent population-based study in Wales suggested that there is currently unmet need for specialist pain services [4]. Therefore, there is a need to understand factors that contribute to the experience of pain within Wales, and to develop healthcare interventions that target modifiable risk factors. This cross-sectional study aims to assess the association between self-reported pain levels and (i) experience of shared-decision-making, (ii) level of trust in the healthcare system, and (iii) health literacy in adults aged 46 years old and over within Wales.

## Methods

### Population health survey

Wales was involved in the Patient-Reported Indicator Survey (PaRIS) [26] for the Organisation for Economic Co-operation and Development (OECD), and collected more responses than the requirement from PaRIS, with this enhanced dataset forming the Population Health Survey [27]. This data provided a national resource to generate evidence at a population level to inform healthcare policy and practice within Wales and was used within the secondary data analysis for this study. Digital Health and Care Wales and Public Health Wales (Value Transformation) are joint data controllers of the dataset.

The survey consisted of complete and partial validated patient-reported outcome measures, patient-reported experience measures, questions from other routine surveys and basic demographics. An international stakeholder group composed of researchers, participating country representatives and the OECD, used systematic reviews and a modified Delphi procedure to develop the survey. As this was an international survey, adaptions were made to ensure it was country specific (e.g., education and ethnicity categories) and translated into local languages. The survey underwent robust field testing. The Population Health Survey for Wales was provided bilingually in English and Welsh, in accordance with the Welsh language (Wales) Measure 2011 [28], with the Welsh translation being undertaken by dedicated bilingual CEDAR researchers.

Further details on the sampling methodology and the OECD PaRIS survey have been reported elsewhere [26]. However, in brief, a stratified random sample of 199 out of 385 GP practices was used, with a subsequent random sample of patients over 45 years old from these selected GP practices, to obtain a sample that was as representative of the Welsh population as possible. The GP practice sampling was undertaken by CEDAR, an NHS research organisation. Although the sampling inclusion criteria was 45 years old and over, the Welsh dataset only contained participants aged 46 years old and over due to an administrative error.

Surveys were completed online, using paper or via telephone, between July-October 2023, with a response rate of 24% (25,838/109,600). A helpline was also provided to give additional language support to participants who required a different language, namely Polish, Bengal, Arabic, and Ukrainian only.

### Outcome – self-reported pain

Level of self-reported pain within the last 7-days prior to the survey completion was ranked on a scale of zero to ten, with zero representing no pain and ten the worst pain imaginable (Appendix 1). The pain variable contained interval data and was treated as a continuous scale.

### Variables of interest

#### Experience of shared decision-making

Participants with chronic health conditions were asked “Are you involved as much as you want about decisions about your care”, with five responses options (Yes, definitely; Yes, to some extent; No, not really; No, definitely not; Not sure).

#### Trust in the healthcare system

All participants were asked “How strongly do you agree or disagree that the healthcare system can be trusted?”, with five response options (strongly agree, agree, neither agree nor disagree, disagree, strongly disagree).

#### Health literacy

The questionnaire contained the 10-item Porter-Novelli Scale (or Consumer Health Information Preferences Scale) which assigns participants into one of four categories detailed by Maibach et al. [29] (independent actives, doctor dependant actives, impendent passives and doctor dependant passives), however, the OECD found three different groups (active engagement, reliance on healthcare professional and patient’s confidence to self-manage) through a factor analysis based on the field trial data. However, as there was a low level of reliability at a country multi-level analysis, the authors concluded that further exploration of these constructs were needed. Instead health literacy was assessed based on the two questions “Most health issues are too complex for me to understand” and “I have difficulty understanding a lot of the health information I read”, as this was the only group that was deemed to be reliable at a country level [30]. A composite measure was created using the two questions, which both had a 5-point scale from strongly disagree to agree. The combined score was categorised into five categories (highest score, above medium, medium score, below medium, lowest score).

#### Explanatory variables

Individual level characteristics included: age in years (46–49, 50–54, 55–59, 60–64, 65–69, 70–74, 75–79, 80–84, 85+), gender (male, female), number of chronic health conditions (1, 2, 3 or more), education (A-level and above, GCSE or equivalent, No qualification), mental health t-score, amount of vigorous or moderate exercise in the past week (none, 1 to 4 days a week, 5–7 days a week) and average alcohol consumption in the past 12 months (not in past 12 months or never, < 1–3 days a month, 1–4 days a week, 5–7 days a week).

Situational/geographic level characteristics included: rurality (city, rural, town/suburbs), local health board and Welsh index of multiple deprivation [WIMD] (20% least deprived, 60–80% most deprived, 40–60% most deprived, 20–40% most deprived, 20% most deprived). Participant WIMD was determine by linking the respondent’s Lower Super Output Area Code to the 2019 WIMD [3], where relative deprivation for Lower Layer Super Output Areas (LSOAs) in Wales are ranked into quintiles from 20% least deprived, to 20% most deprived.

### Data analysis

All data cleaning and statistical analyses were completed in R Studio (R studio version 2024.12.1–563: R version 4.4.3) [31]. Descriptive statistics included mean and standard deviation (SD) or median and interquartile range (IQR) for continuous variables, and counts (n) and percentage (%) for categorical variables. Due to the clustering of the dataset of patients, within GP surgeries, within health boards, a multilevel linear regression was considered, adjusting for individual and situational characteristics to assess the association between the level of self-reported pain within the last seven days prior to the survey and (i) shared decision-making, (ii) health literacy, and (iii) trust in the healthcare system. However, on investigation the level of clustering within the resultant data sample was small, with an intraclass correlation coefficient (ICC) of < 0.001 in the model with a random effect for health boards and GP surgeries, and with a random effect just for health boards. Therefore, a single level linear regression model was used, with health board included as a confounding factor. Due to the potential moderating effect of gender, a stratified sub-analysis by gender was also undertaken. The lm() function in the stats package [31] was used for the linear regression model, and collinearity was checked using the vif() function in the Car package [32]. Missing values were dealt with through case-wise deletion and no attempt was made to weight the data. Statistical significance was defined as a *p* value below 0.05 and a 95% confidence interval that does not cross zero.

## Results

### Sample description

Out of the 25,839 responders to the survey, 18,909 had at least one chronic health condition, which form the final sample for this analysis. The sample had a median pain score of 4 (IQR: 1–6) (Table 1). The majority of responders felt that they were involved in decision about their care as much as they wanted to be, either fully (34.1%) or to some extent (36.8%). The largest proportion of responders neither agreed nor disagreed (36.1%) that the healthcare system can be trusted, followed closely by agree (35.2%). An above middle health literacy skill was observed for the greatest proportion of responders (41.1%).

**Table 1:** Descriptive statistics of responders with chronic health condition (*N* = 18,909)

	*n* (%)
**Self-reported average pain for the last 7-days**	
Mean (SD)	3.71 (2.7)
Median (IQR)	4 (1–6)
*Missing*	*35 (0.1)*
**Are you involved as much as you want to be in decisions about your care?**	
Yes, definitely	6447 (34.1)
Yes, to some extent	6963 (36.8)
No, not really	3269 (17.3)
No, definitely not	997 (5.3)
Not sure	905 (4.8)
*Missing*	*328 (1.7)*
**How strongly do you agree or disagree that the healthcare system can be trusted?**	
Strongly disagree	919 (4.9)
Disagree	2032 (10.8)
Neither agree nor disagree	6818 (36.1)
Agree	6659 (35.2)
Strongly agree	1700 (9.0)
*Missing*	*781 (4.1)*
**Health literacy (Combined)**	
Highest score	3067 (16.2)
Above middle	7772 (41.1)
Middle score	5486 (29.0)
Below middle	2126 (11.2)
Lowest score	356 (1.9)
*Missing*	*102 (0.5)*
**Age (years)**	
46–49	898 (4.8)
50–54	1712 (9.1)
55–59	2319 (12.3)
60–64	2807 (14.9)
65–69	3133 (16.6)
70–74	3138 (16.6)
75–79	2712 (14.3)
80–84	1298 (6.9)
85+	866 (4.6)
*Missing*	*26 (0.1)*
**Gender**	
Female	9522 (50.4)
Male	8751 (46.3)
Other	636 (3.4)
**Number of chronic health conditions**	
1	7733 (40.9)
2	5758 (30.5)
3 plus	5418 (28.7)
**Occupation**	
Employed	5695 (30.1)
Other	737 (3.9)
Retired	10,702 (56.6)
Unable to work due to sickness or ill-health	1156 (6.1)
*Missing*	*619 (3.3)*
**Education**	
No formal qualifications	3452 (18.3)
GCSE, NVQ or Equivalent	4538 (24.0)
A-level, degree, masters of equivalent	9197 (48.0)
*Missing*	*1722 (9.1)*
**Mental health** ***t*-score**	
Mean (SD)	46.41 (9.1)
Median (IQR)	45.8 (41.4–53.5)
*Missing*	*105 (0.6)*
**In the past week**,** on how many days did you do at least 30 min of either vigorous or moderate activity?**	
None	7274 (38.5)
1 to 4 days a week	7091 (37.5)
5–7 days a week	4108 (21.7)
Missing	436 (2.3)
**In the past 12 months**,** how often have you had an alcoholic drink of any kind?**	
Not in past 12 months or never	3361 (17.8)
< 1–3 days a month	5819 (30.8)
1–4 days a week	6666 (35.3)
5–7 days a week	3023 (16.0)
*Missing*	*40 (0.2)*
**Area**	
City	2407 (12.7)
Rural	7352 (38.9)
Town or suburb	8364 (44.2)
*Missing*	*786 (4.2)*
**Deprivation quintile**	
20% least deprived	4251 (22.5)
20–40% most deprived	3169 (16.8)
40–60% most deprived	4088 (21.6)
60–80% most deprived	5042 (26.7)
20% least deprived	2350 (12.4)
*Missing*	*9 (0.1)*
**Health board**	
Health board 1	3483 (18.4)
Health board 2	4734 (25.0)
Health board 3	2958 (15.6)
Health board 4	2038 (10.8)
Health board 5	2779 (14.7)
Health board 6	929 (4.9)
Health board 7	1983 (10.5)
*Missing*	*5 (0.03)*

### Experience of shared-decision-making

**Fig. 1:**
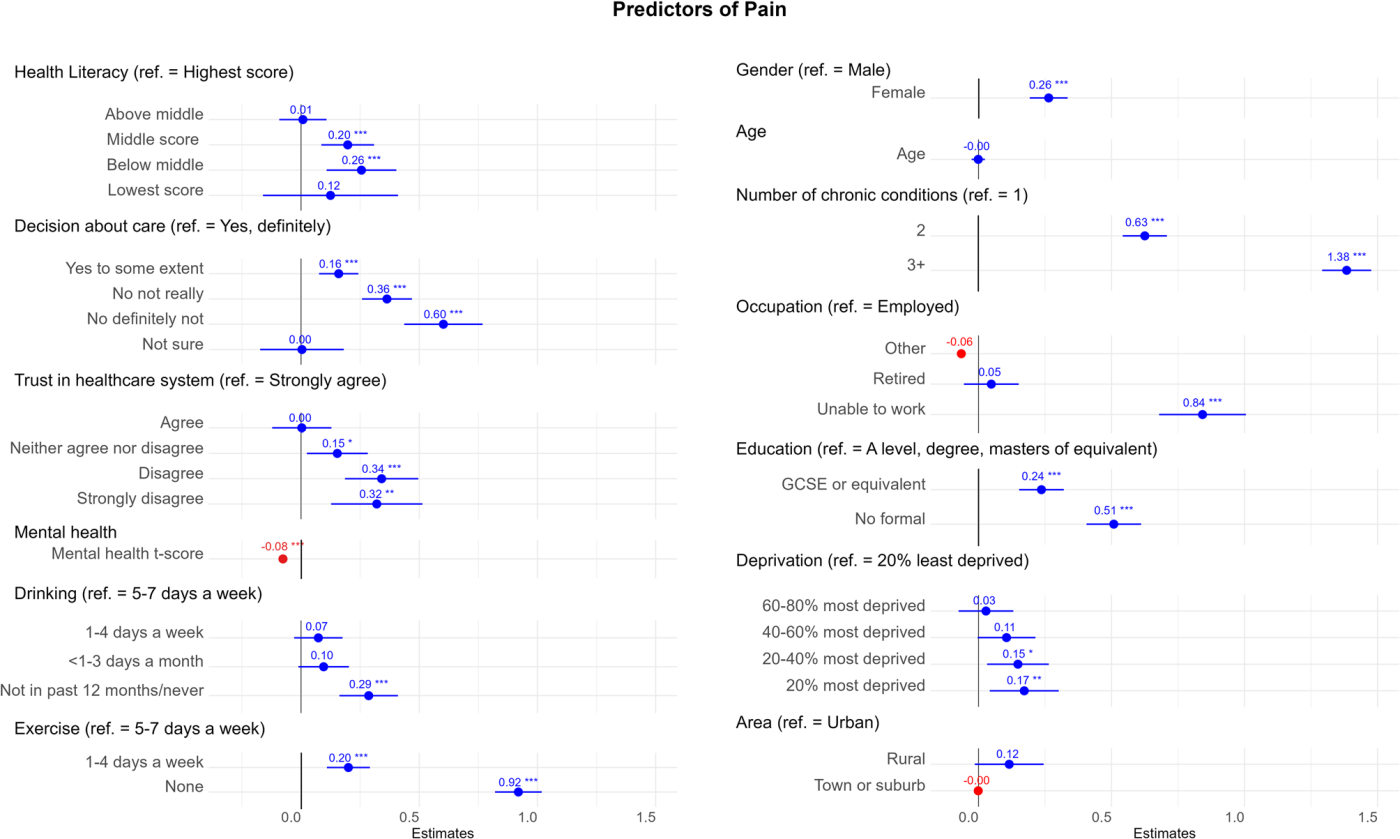
Forest plot of the adjusted regression estimates (***B*** and 95% confidence interval) for the predictors of pain for all variables included in final model (*N* = 16,197). Model fit statistics: R^2^ = 0.319, AIC = 71769.13. (Abbreviations: Ref. = reference category, *** = <0.001, ** = 0.001, * = 0.05). Tabulated results in Appendix 2

An association was observed between decreasing experience of shared decision-making decreased and increasing pain score. Compared to individuals who definitely felt that they were involved as much as they wanted about decisions in their care, those only being involved to some extent (*B* = 0.16; 95% CI: 0.08–0.24), not really (*B* = 0.36; 95% CI: 0.26–0.47) and definitely not (*B* = 0.60; 95% CI: 0.44–0.77) showed a statistically significant increase in pain score (Fig. 1).

### Health literacy

The combined health literacy score was observed to have some association with pain score. Compared to individuals with the highest health literacy score, individuals with a middle health literacy score (*B* = 0.20; 95% CI: 0.09–0.31) or below middle health literacy score (*B* = 0.26; 95% CI: 0.11–0.40) had a statistically significant increase in pain score. No associations were observed with the lowest and above middle health literacy score (Fig. 1).

### Perception of the healthcare system

An association was observed between decreasing trust in the healthcare system and increasing pain scores. Compared to those strongly agreeing that the healthcare system can be trusted, those agreeing (*B* = 0.15; 95% CI: 0.02–0.28), neither agreeing nor disagreeing (*B* = 0.34; 95% CI: 0.19–0.50), disagreeing (*B* = 0.32; 95% CI: 0.13–0.51) and strongly disagreeing (*B* = 0.32; 95% CI: 0.13–0.51) that the healthcare system can be trusted, had a statistically significant increase in pain score (Fig. 1).

### Other associated variables

Individual level factors that were observed to be associated with increased pain included: identifying as female (*B* = 0.26; 95% CI: 0.19–0.34); having two (*B* = 0.63; 95% CI: 0.54–0.71) or three or more chronic health conditions (*B* = 1.38; 95% CI: 1.29–1.48) compared to one chronic health condition; being unable to work due to sickness or ill-health (*B* = 0.84; 95% CI: 0.68–1.01), an education level of GCSE/NVQ or equivalent (*B* = 0.24; 95% CI: 0.15–0.32) or no formal qualifications (*B* = 0.51 ; 95% CI: 0.41–0.61) compared to having a qualification of A-level or above; not drinking in the past month or ever (*B* = 0.20; 95% CI: 0.11–0.29); and exercising 1–4 days a week (*B* = 0.29; 95% CI: 0.16–0.41) or not exercising (*B* = 0.20; 95% CI: 0.11–0.29) compared to exercising 5–7 days a week. However, having a higher mental health t-score was observed to be associated with a decrease, with a one-point increase in the mental health t-score reducing the pain score by 0.08 (*B* = -0.08; 95% CI: -0.08 – -0.07) (Fig. 1).

Deprivation level was the only situational level factor found to be associated with pain, with the 20–40% most deprived (*B* = 0.15; 95% CI: 0.03–0.26) and the 20% most deprived (*B* = 0.17; 95% CI: 0.04–0.30) quintiles having an increase in pain score compared to the 20% least deprived quintile (Fig. 1).

### Sub-analysis – females only

In females, a decrease in shared decision-making and a decrease in trust in the healthcare system was associated with an increasing pain score (Appendix 3). Compared to individuals who definitely felt that they were involved as much as they wanted about decision in their care, those only being involved to some extent (*B* = 0.15; 95% CI: 0.04–0.27), not really (*B* = 0.36; 95% CI: 0.21–0.51) and definitely not (*B* = 0.77; 95% CI: 0.54–1.01) showed a statistically significant increase in pain score. Compared to those strongly agreeing that the healthcare system can be trusted, those disagreeing (*B* = 0.26; 95% CI: 0.03–0.49) and strongly disagreeing (*B* = 0.36; 95% CI: 0.08–0.65) that the healthcare system can be trusted had a statistically significant increase in pain score. The only relevant association observed with health literacy was an increase in pain for individuals with a middle health literacy score compared to the highest health literacy score (*B* = 0.19; 95% CI: 0.04–0.35).

### Sub-analysis – males only

In males, a decrease in shared decision-making and a decrease in trust in the healthcare system was associated with an increasing pain score (Appendix 3). Compared to individuals who definitely felt that they were involved as much as they wanted about decision in their care, those only being involved to some extent (*B* = 0.16; 95% CI: 0.04–0.27), not really (*B* = 0.35; 95% CI: 0.20–0.50) and definitely not (*B* = 0.40; 95% CI: 0.16–0.64) showed a statistically significant increase in pain score. Compared to those strongly agreeing that the healthcare system can be trusted, those neither agreeing nor disagreeing (*B* = 0.28; 95% CI: 0.01–0.54), disagreeing (*B* = 0.43; 95% CI: 0.22–0.65) and strongly disagreeing (*B* = 0.19; 95% CI: 0.01–0.36) that the healthcare system can be trusted had a statistically significant increase in pain score. Two associations were observed with health literacy with an increase in pain for individuals with a middle health literacy score (*B* = 0.19; 95% CI: 0.04–0.35) and below middle health literacy score (*B* = 0.32; 95% CI: 0.11–0.53) compared to the highest health literacy score.

## Discussion

This population-based study showed that individuals aged 46 years old and over in Wales with at least one chronic health condition who self-reported greater levels of pain also reported lower levels of shared-decision-making in their care, health literacy and trust in the healthcare system. Although these associations were statistically significant, the clinical significance of magnitude of change in pain levels could not be fully interpreted, due to the variety of thresholds present within the literature that were specific to setting and type of pain. However, in the context of wider chronic disease management, any small change in pain level could be beneficial and clinically meaningful, when looking at the bigger picture; but this requires further exploration. Despite this, these results have implications for the equitable delivery of chronic disease management in Wales to ensure that patients are adequately managed in a way that matters most to them, and meets the Welsh Government’s Value-Based Healthcare approach.

The association between shared decision-making and chronic pain is well documented within the literature [22, 33], which corresponds with the results within this study. There is the potential that increasing the level of shared decision-making, even to the point where shared decision-making is experienced to “some extent”, may support the reduction of pain, as the confidence intervals do not overlap. Despite there being evidence within the literature that improving shared decision-making can influence the level of chronic pain experience [22], the interpretation of the results of this study should be read with caution as the directionality of the effect cannot be determined within this analysis. Similarly, a decrease in health literacy is associated within an increase in self-reported pain, which is supported by findings within the literature [34, 35]. The fact that the lowest health literacy score is not associated in these results may be an artefact of the proportionally low numbers of observations within this category (Appendix 2). Low numbers of observations were also seen in the extremities of the 5-point scale for the trust in healthcare system (i.e., strongly agree and strongly disagree) which may explain the similarity in effect estimate between “strongly agree” and “agree”, and “strongly disagree” and “disagree”. Nevertheless, self-reported pain is associated with trust in the healthcare systems within this study and within the literature [36, 37]. Even though associations were observed, any chronic pain intervention (e.g., decision aids, upskilling staff) to increase shared-decision-making, health literacy and trust in the healthcare system, may not produce clinically meaningful changes in pain; therefore, future high-quality interventional studies are required. Despite the lack of a clinically meaningful change, any reduction in pain levels could be beneficial and there are wider potential benefits to other health outcomes, and to the health service, which require further exploration.

The increased risk of pain and number of chronic health conditions is well documented within the literature [14], and within this study is the leading risk factor for self-reported pain. As number of chronic health conditions is a non-modifiable risk factor, it could be used as risk stratification tools within the implementation of interventions, however, this would require further research to understand the implication of such a strategy. Gender is another non-modifiable risk factor and is associated with level of chronic pain within this study and the literature, the mechanism is more complicated and is influenced by gender roles, ability to cope with pain [38] and differences in perception [39]. In the sub-analyses by gender some differences were observed in the results, however, no substantive conclusions could be drawn as the confidence intervals overlapped. The association of lower exercise with increased perception of pain, maybe as a results of pain preventing exercise, however directionality of the effect cannot be determined. Encouraging positive health behaviours and active self-management strategies (e.g., increasing exercise) and increasing workforce participation through adoption of flexible working policies could be beneficial within chronic pain management [9, 40] but would also have wider health benefits [41, 42].

Upskilling of staff in shared-decision making is already underway in Wales, through Health Education and Improvement Wales (HEIW). However, perception, expression and impact of pain is intrinsically linked to the social determinants of health, which is demonstrated within the results of this study, providing confidence in our results, but also demonstrating the need to understand the multilevel social context when completing biopsychosocial pain assessments [17]. As inequalities persist, any interventions to increase shared decision-making, trust in healthcare system and health literacy within the healthcare system should be mindful of the social determinants of health and take an approach that includes multilevel interdisciplinary and intersectoral collaborations [17].

The results of this study are based on a large scale nationally representative questionnaire, which was comparable to other nationally held demographic data sets. However, it is cross-sectional in design and therefore only represents one time point and is subject to response and recall biases. The response to the pain questions is self-reported based on the last seven days, but is based on a validated question. As there was only one pain question within the questionnaire, chronic and acute pain could not be differentiated, therefore any interpretation and conclusions are based on general pain, not specifically chronic pain. However, the 3-month cut off for chronic pain has been described as being arbitrary [43] and the sample is likely to be dominated by chronic pain. In addition, the questionnaire captured gender, not the biological sex of the responder, which limits the ability to comment on the biological plausibility of the relationship with pain. However, it is likely that this would affect a very small proportion of the observations included in the analysis, especially as observations with a report of “Non-binary”, “Prefer to self-describe” or “Other” were excluded due to small numbers, even after combining into one category. Similarly, ethnicity could not be controlled for within the analysis due to the low numbers in all groups other than White ethnicity. Finally, case-wise deletion was used to deal with missing data, as there was a low proportion of missing data (Table 1). This approach is unlikely to have significantly biased the data but may have reduced the statistical power due to a reduction in the sample size.

## Conclusion

Pain levels experienced by people aged 46 years old and over with at least one chronic health condition are associated with their experience of shared decision-making, health literacy level and perception of the healthcare system. The Welsh healthcare service should actively consider ways to improve the role of share decision-making, trust in the health care system and increase health literacy level within the management of chronic pain. Taking a person-centred approach and understanding what matters most to patients, not only has the potential to improve pain levels among people with chronic health conditions, but it would align with the NICE guidelines for chronic pain management, and the Welsh Government’s Value-Based Healthcare approach.

## Supplementary Information

Below is the link to the electronic supplementary material.

Supplementary Material 1

## Data Availability

The data are not publicly available due to privacy or ethical restrictions.

## Abbreviations

95% CI: 95% confidence interval
CEDAR: Centre for Healthcare Evaluation Device Assessment and Research
GCSE: General Certificate of Secondary Education
GP: General Practitioner
IQR: interquartile range
NHS: National Health Service
NICE: National Institute of Clinical Evidence
NVQ: National Vocational Qualification
OECD: Organisation for Economic Co–operation and Development
PaRIS: Patient–Reported Indicator Surveys
SD: standard deviation
SDM: Share decision–making
WIMD: Welsh Index of Multiple Deprivation
LSOAs: Lower Layer Super Output Areas
