# Supplementary material for: Shared-decision-making, trust in the healthcare system and health literacy are associated with self-reported pain levels: a population-based cross-sectional study in individuals living with a chronic health condition in Wales
Source: BMC Health Serv Res. 2025 Dec 1;25:1556. doi: 10.1186/s12913-025-13724-3 (PMC12667160; doi:10.1186/s12913-025-13724-3)
Supplement: Supplementary file 1 — Supplementary Material 1 [file 12913_2025_13724_MOESM1_ESM.docx]

**Appendix**

Appendix 1: survey question for self-reported pain level


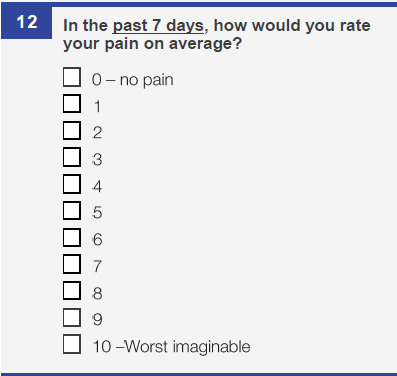


Appendix 2: Adjusted regression estimates (B and 95 % confidence interval) for the predictors of pain.

|  | **Model sample after account for missing data (N=16,194)** | **Adjusted Estimate (95% CI)** | ***p* value** |
| --- | --- | --- | --- |
|  | **n (%)** |  |  |
| **Self-reported average pain for the last 7-days [outcome]** | | | |
| Mean (SD) | 3.34 (2.68) | . | . |
| Median (IQR) | 3 (1-6) | . | . |
| **Are you involved as much as you want to be in decisions about your care?** | | | |
| Yes, definitely | 5668 (35) | *Ref.* |  |
| Yes, to some extent | 6095 (37.64) | **0.16 (0.08 – 0.24)** | **<0.001** |
| No, not really | 2849 (17.59) | **0.63 (0.26 – 0.47)** | **<0.001** |
| No, definitely not | 874 (5.40) | **0.60 (0.44 – 0.77)** | **<0.001** |
| Not sure | 708 (4.37) | 0.00 (-0.17 – 0.18) | 0.965 |
| **How strongly do you agree or disagree that the healthcare system can be trusted?** | | | |
| Strongly disagree | 817 (5.05) | **0.32 (0.13 – 0.51)** | **0.001** |
| Disagree | 1832 (11.31) | **0.34 (0.19 – 0.50)** | **<0.001** |
| Neither agree nor disagree | 5935 (36.65) | **0.15 (0.02 – 0.28)** | **0.020** |
| Agree | 6057 (37.4) | 0.00 (-0.12 – 0.13) | 0.959 |
| Strongly agree | 1553 (9.59) | *Ref.* |  |
| **Health literacy (Combined)** |  |  |  |
| Highest score | 2792 (17.24) | *Ref.* |  |
| Above middle | 6929 (42.79) | 0.01 (-0.09 – 0.11) | 0.875 |
| Middle score | 4574 (28.25) | **0.20 (0.09 – 0.31)** | **0.001** |
| Below middle | 1622 (10.02) | **0.26 (0.11 – 0.40)** | **0.001** |
| Lowest score | 277 (1.71) | 0.12 (-0.16 – 0.41) | 0.392 |
| **Age (years)** |  |  |  |
| 46-49 | 811 (5.01) | -0.00 (-0.03 – 0.02) | 0.953 |
| 50-54 | 1524 (9.41) |  |  |
| 55-59 | 2065 (12.75) |  |  |
| 60-64 | 2498 (15.43) |  |  |
| 65-69 | 2750 (16.98) |  |  |
| 70-74 | 2684 (16.57) |  |  |
| 75-79 | 2241 (13.84) |  |  |
| 80-84 | 985 (6.08) |  |  |
| 85+ | 636 (3.93) |  |  |
| **Gender** |  |  |  |
| Female | 8297 (51.24) | **0.26 (0.19 – 0.34)** | **<0.001** |
| Male | 7897 (48.76) | *Ref.* |  |
| Other |  |  |  |
| **Number of chronic health conditions** |  |  |  |
| 1 | 6660 (41.13) | *Ref.* |  |
| 2 | 4933 (30.46) | **0.63 (0.54 – 0.71)** | **<0.001** |
| 3 plus | 4601 (28.41) | **1.38 (1.29 – 1.48)** | **<0.001** |
| **Occupation** |  |  |  |
| Employed | 5342 (32.99) | *Ref.* |  |
| Other | 603 (3.72) | -0.06 (-0.25 – 0.12) | 0.500 |
| Retired | 9249 (57.11) | 0.05 (-0.05 – 0.15) | 0.360 |
| Unable to work due to sickness or ill-health | 1000 (6.18) | **0.84 (0.68 – 1.01)** | **<0.001** |
| **Education** |  |  |  |
| No formal qualifications | 3081 (19.03) | **0.51 (0.41 – 0.61)** | **<0.001** |
| GCSE, NVQ or Equivalent | 4240 (26.18) | **0.24 (0.15 – 0.32)** | **<0.001** |
| A level, degree, masters of equivalent | 8873 (54.79) | *Ref.* |  |
| **Mental health t-score** |  |  |  |
| Mean (SD) | 46.64 (9.08) | **-0.08 (-0.08 – 0.07)** | **<0.001** |
| Median (IQR) | 45.8 (41.4-53.5) |  |  |
| **In the past week, on how many days did you do at least 30 minutes of either vigorous or moderate activity?** | | | |
| None | 6161 (38.47) | **0.92 (0.82 – 1.02)** | **<0.001** |
| 1 to 4 days a week | 6354 (37.50) | **0.20 (0.11 – 0.29)** | **<0.001** |
| 5-7 days a week | 3679 (21.72) | *Ref.* |  |
| **In the past 12 months, how often have you had an alcoholic drink of any kind?** | | | |
| Not in past 12 months or never | 2656 (16.4) | **0.29 (0.16 – 0.41)** | **<0.001** |
| <1-3 days a month | 4963 (30.65) | 0.10 (-0.01 – 0.20) | 0.079 |
| 1-4 days a week | 5897 (36.41) | 0.07 (-0.03 – 0.18) | 0.160 |
| 5-7 days a week | 2678 (16.54) | *Ref.* |  |
| **Area** |  |  |  |
| City | 2161 (13.34) | *Ref.* |  |
| Rural | 6532 (40.34) | 0.12 (-0.01 – 0.24) | 0.081 |
| Town or suburb | 7501 (46.32) | -0.00 (-0.12 – 0.12) | 0.985 |
| **Deprivation quintile** |  |  |  |
| 20% most deprived | 1895 (11.70) | **0.17 (0.04 – 0.30)** | **0.009** |
| 20-40% most deprived | 2677 (16.53) | **0.15 (0.03 – 0.26)** | **0.012** |
| 40-60% most deprived | 3483 (21.51) | 0.11 (-0.00 – 0.21) | 0.058 |
| 60-80% most deprived | 4391 (27.11) | 0.03 (-0.07 – 0.13) | 0.594 |
| 20% least deprived | 3748 (23.14) | *Ref.* |  |
| **Health board** |  |  |  |
| Health board 1 | 2975 (18.37) | 0.05 (-0.06 – 0.18) | 0.483 |
| Health board 2 | 4044 (24.97) | -0.02 (-0.14 – 0.11) | 0.789 |
| Health board 3 | 2566 (15.85) | *Ref.* |  |
| Health board 4 | 1703 (10.52) | 0.05 (-0.08 – 0.18) | 0.483 |
| Health board 5 | 2365 (14.6) | 0.04 (-0.07 – 0.16) | 0.459 |
| Health board 6 | 838 (5.17) | -0.10 (-0.27 – 0.07) | 0.241 |
| Health board 7 | 1703 (10.52) | **0.15 (0.02 – 0.29)** | **0.026** |
| Model fit statistics: R2 = 0.319, AIC = 71769.13  Abbreviations: Ref. = reference category, 95% CI = 95% confidence interval | | | |

Appendix 3: Sub-analysis Adjusted regression estimates (B and 95 % confidence interval) for the predictors of pain by gender.

|  | **Females (N=8297)** | | **Males (N=7897)** | |
| --- | --- | --- | --- | --- |
|  | **Adjusted estimate (95% CI)** | ***p* value** | **Adjusted estimate (95% CI)** | ***p* value** |
| **Are you involved as much as you want to be in decisions about your care?** | | | |  |
| Yes, definitely | *Ref.* |  | *Ref.* |  |
| Yes, to some extent | **0.15 (0.04 – 0.27)** | **0.009** | **0.16 (0.04 – 0.27)** | **0.010** |
| No, not really | **0.36 (0.21 – 0.51)** | **<0.001** | **0.35 (0.20 – 0.50)** | **<0.001** |
| No, definitely not | **0.77 (0.54 – 1.01)** | **<0.001** | **0.40 (0.16 – 0.64)** | **0.001** |
| Not sure | -0.05 (-0.21 – 0.18) | 0.856 | 0.06 (-0.20 – 0.32) | 0.662 |
| **How strongly do you agree or disagree that the healthcare system can be trusted?** | | | |  |
| Strongly disagree | **0.36 (0.08 – 0.65)** | **0.013** | **0.28 (0.01 – 0.54)** | **0.039** |
| Disagree | **0.26 (0.03 – 0.49)** | **0.013** | **0.43 (0.22 – 0.65)** | **<0.001** |
| Neither agree nor disagree | 0.12 (-0.08 – 0.31) | 0.235 | **0.19 (0.01 – 0.36)** | **0.035** |
| Agree | -0.02 (-0.21 – 0.18) | 0.856 | 0.01 (-0.16 – 0.17) | 0.925 |
| Strongly agree | *Ref.* |  | *Ref.* |  |
| **Health literacy (Combined)** |  |  |  |  |
| Highest score | *Ref.* |  | *Ref.* |  |
| Above middle | -0.01 (-0.04 – 0.13) | 0.926 | 0.04 (-0.11 – 0.19) | 0.622 |
| Middle score | **0.19 (0.04 – 0.35)** | **0.013** | **0.22 (0.05 – 0.38)** | **0.009** |
| Below middle | 0.20 (-0.01 – 0.41) | 0.061 | **0.32 (0.11 – 0.53)** | **0.003** |
| Lowest score | 0.24 (-0.16 – 0.64) | 0.234 | -0.00 (-0.41 – 0.41) | 0.996 |
| **Age (years)** | -0.00 (-0.04 – 0.03) | 0.889 | 0.00 (-0.03 – 0.04) | 0.938 |
| **Number of chronic health conditions** |  |  |  |  |
| 1 | *Ref.* |  | *Ref.* |  |
| 2 | 0.64 (0.52 – 0.75) | **<0.001** | **0.61 (0.49 – 0.73)** | **<0.001** |
| 3 plus | 1.45 (1.32 – 1.58) | **<0.001** | **1.31 (1.18 – 1.44)** | **<0.001** |
| **Occupation** |  |  |  |  |
| Employed | *Ref.* |  | *Ref.* |  |
| Other | -0.06 (-0.30 – 0.17) | 0.613 | -0.02 (-0.24 – 0.30) | 0.925 |
| Retired | 0.12 (-0.03 – 0.27) | 0.113 | -0.01 (-0.15 – 0.13) | 0.893 |
| Unable to work due to sickness or ill-health | **0.85 (0.63 – 1.07)** | **<0.001** | **0.81 (0.57 – 1.06)** | **<0.001** |
| **Education** |  |  |  |  |
| No formal qualifications | **0.39 (0.20 – 0.56)** | **<0.001** | **0.61 (0.48 – 0.75)** | **<0.001** |
| GCSE, NVQ or Equivalent | **0.14 (0.02 – 0.26)** | **0.017** | **0.35 (0.22 – 0.47)** | **<0.001** |
| A level, degree, masters of equivalent | *Ref.* |  | *Ref.* |  |
| **Mental health t-score** | **-0.08 (-0.09** – **-0.07)** | **<0.001** | **-0.07 (-0.08** – **-0.07)** | **<0.001** |
| **In the past week, on how many days did you do at least 30 minutes of either vigorous or moderate activity?** | | | | |
| None | 0.93 (0.79 – 1.07) | **<0.001** | **0.92 (0.78 – 1.06)** | **<0.001** |
| 1 to 4 days a week | 0.27 (0.14 – 0.40) | **<0.001** | 0.13 (0.01 – 0.26) | 0.086 |
| 5-7 days a week | *Ref.* |  | *Ref.* |  |
| **In the past 12 months, how often have you had an alcoholic drink of any kind?** | | | |  |
| Not in past 12 months or never | 0.38 (.20 – 0.56) | **<0.001** | 0.15 (-0.02 – 0.33) | 0.086 |
| <1-3 days a month | 0.12 (-0.04 – 0.28) | 0.147 | 0.09 (-0.06 – 0.24) | 0.229 |
| 1-4 days a week | 0.09 (-0.08 – 0.25) | 0.296 | 0.07 (-0.06 – 0.20) | 0.283 |
| 5-7 days a week | *Ref.* |  | *Ref.* |  |
| **Area** |  |  |  |  |
| City | *Ref.* |  | *Ref.* |  |
| Rural | 0.13 (-0.05 – 0.32) | 0.154 | 0.09 (-0.13 – 0.27) | 0.339 |
| Town or suburb | 0.02 (-0.14 – 0.19) | 0.798 | -0.03 (-0.19 – 0.14) | 0.774 |
| **Deprivation quintile** |  |  |  |  |
| 20% most deprived | **0.28 (0.10 – 0.46)** | **0.003** | 0.06 (-0.13 – 0.24) | 0.531 |
| 20-40% most deprived | 0.15 (-0.01 – 0.31) | 0.071 | 0.14 (-0.02 – 0.31) | 0.089 |
| 40-60% most deprived | **0.24 (0.09 – 0.39)** | **0.002** | -0.04 (-0.20 – 0.11) | 0.600 |
| 60-80% most deprived | 0.07 (-0.08 – 0.21) | 0.374 | -0.00 (-0.15 – 0.14) | 0.956 |
| 20% least deprived | *Ref.* |  | *Ref.* |  |
| **Health board** |  |  |  |  |
| Health board 1 | 0.00 (-0.15 – 0.15) | 0.997 | 0.09 (-0.07 – 0.25) | 0.26 |
| Health board 2 | -0.08 (-0.26 – 0.10) | 0.380 | 0.04 (-0.14 – 0.22) | 0.696 |
| Health board 3 | *Ref.* |  | *Ref.* |  |
| Health board 4 | 0.04 (-0.14 – 0.23) | 0.635 | 0.04 (-0.14 – 0.23) | 0.637 |
| Health board 5 | 0.01 (-0.15 – 0.17) | 0.865 | 0.07 (-0.09 – 0.23) | 0.408 |
| Health board 6 | **-0.27 (-0.51 – -0.03)** | **0.025** | 0.06 (-0.17 – 0.30) | 0.603 |
| Health board 7 | 0.10 (-0.09 – 0.28) | 0.322 | **0.22 (0.03 – 0.41)** | **0.022** |
| Model fit statistics: Females only - R^2^ = 0.327, Males only – R^2^ = 0.305  Abbreviations: Ref. = reference category, 95% CI = 95% confidence interval | | | | |
